# The Roles of Exosomes as Future Therapeutic Agents and Diagnostic Tools for Glioma

**DOI:** 10.3389/fonc.2021.733529

**Published:** 2021-10-13

**Authors:** Xiaoben Wu, Xingbang Wang, Jing Wang, Yingying Hao, Fang Liu, Xin Wang, Lei Yang, Zhiming Lu

**Affiliations:** ^1^ Department of Clinical Laboratory, Shandong Provincial Hospital Affiliated to Shandong First Medical University, Jinan, China; ^2^ Department of Neurology, Qilu Hospital, Shandong University, Jinan, China; ^3^ Department of Medical Engineering, Shandong Provincial Hospital Affiliated to Shandong First Medical University, Jinan, China

**Keywords:** exosomes, glioma, diagnostic biomarker, prognostic biomarker, treatment

## Abstract

Glioma is a common type of tumor originating in the brain. Glioma develops in the gluey supporting cells (glial cells) that surround and support nerve cells. Exosomes are extracellular vesicles that contain microRNAs, messenger RNA, and proteins. Exosomes are the most prominent mediators of intercellular communication, regulating, instructing, and re-educating their surrounding milieu targeting different organs. As exosomes’ diameter is in the nano range, the ability to cross the blood–brain barrier, a crucial obstacle in developing therapeutics against brain diseases, including glioma, makes the exosomes a potential candidate for delivering therapeutic agents for targeting malignant glioma. This review communicates the current knowledge of exosomes’ significant roles that make them crucial future therapeutic agents and diagnostic tools for glioma.

## 1 Introduction

Gliomas are the highest known primary malignant tumor that affects the brain. Among all the gliomas, glioblastoma (GBM) is the most prevalent type among all other types. It occupies 70% of all gliomas and has a median overall survival of 15 months (1). The United States’ incident rate is 3.20 per 100,000, and GBM handles 60%–70% of malignant glioma (2, 3). Glioma is the third highest cause of cancer deaths in patients aged 15 and 34 years, which is responsible for 2.5% of the worldwide cancer fatality rate. Glioblastoma multiforme is 50% of gliomas, with a higher prevalence of patients over 65 years of age (4).

Multiple occurrences such as high growth rate, widespread invasion, and genetic variations are characteristics of gliomas. The drug resistance of GBM, the poor prognosis of patients who harbor glioma, is significantly linked to the lack of insight into the molecular facts related to the initiation of glioma and the absence of sensitive diagnosis and accurate therapeutic agents (5). The glioma microenvironment comprises different cells other than tumor cells, such as astrocytes, microglia, endothelial cells, and immune cells. Currently, trimetazidine (TMZ) is the primary chemotherapy drug for GBM (6). Patients with GBM have relatively high treatment resistance, resulting in decreasing overall survival (7). Immunotherapy inhibits the immune checkpoint receptor of programmed cell protein 1 (PD-1), and bevacizumab that inhibits the vascular endothelial growth factor is now being studied to enhance the treatment outcome of patients treated with GBM, which is the most prevalent glioma type (8). The determination of GBM is primarily focused on imaging methods and biopsies of tissues. However, imaging methods cannot reliably distinguish lesions induced by tumor development from treatment-related pseudo-progression lesions that mimic tumor progression and may typically resolve with time (9). Liquid biopsies allow identifying circulating biomarkers and offer the advantage of being non-invasive, thus enabling serial sampling and tracking possible structural reforms in the tumor during therapy (10).

At present, the critical testing techniques for diagnosing gliomas are based on neurological tests and neuroimaging procedures are sometimes performed when gliomas are at the advanced level (11). Exosomes are extracellular vesicles first identified 30 years ago. They have since been connected to cell–cell communication, disease propagation, and drug development (12). Those exosomes comprise different bioactive molecules, such as microRNAs (miRNAs), messenger RNA (mRNA), and other vital protein compounds (13).

Exosomes are critical for cellular signaling in normal physiology and pathological conditions, most notably in cancer. Exosomes are potent progenitors capable of changing target cell phenotypes, most notably during carcinogenesis and development, by altering tumor microenvironments and assisting in establishing the pre-metastatic niche. Numerous features of exosomes point to them as a new method for identifying cancer biomarkers for early diagnosis and therapeutic targets and for utilizing exosomes’ inherent and modified properties as therapeutic tools to slow down disease development (14). The blood–brain barrier (BBB) protects the central nervous system, supplies nourishment, regulates homeostasis, and allows the brain and the rest of the human body to communicate through the serum. Because of the complex structure of the BBB, drug administration in the brain is a significant problem, necessitating the discovery of innovative methods to achieve improved drug delivery in the brain, either invasively or non-invasively (15). To solve the issue mentioned above, different studies have been proposed to use exosomes to deal with this issue due to their nano size. Also, various studies revealed that exosomes are highly or lowly expressed in different cancers, including glioma. In addition, exosomes are also involved in other main cancer events, such as cancer initiation and progression of various cancers (16); however, this active status made exosomes potential therapeutic and biomarkers tools for glioma (17). The primary purpose of this review is to highlight the roles of exosomes as future therapeutic agents and diagnostic tools for glioma

## 2 Exosome’s Biology

**Figure 1:**
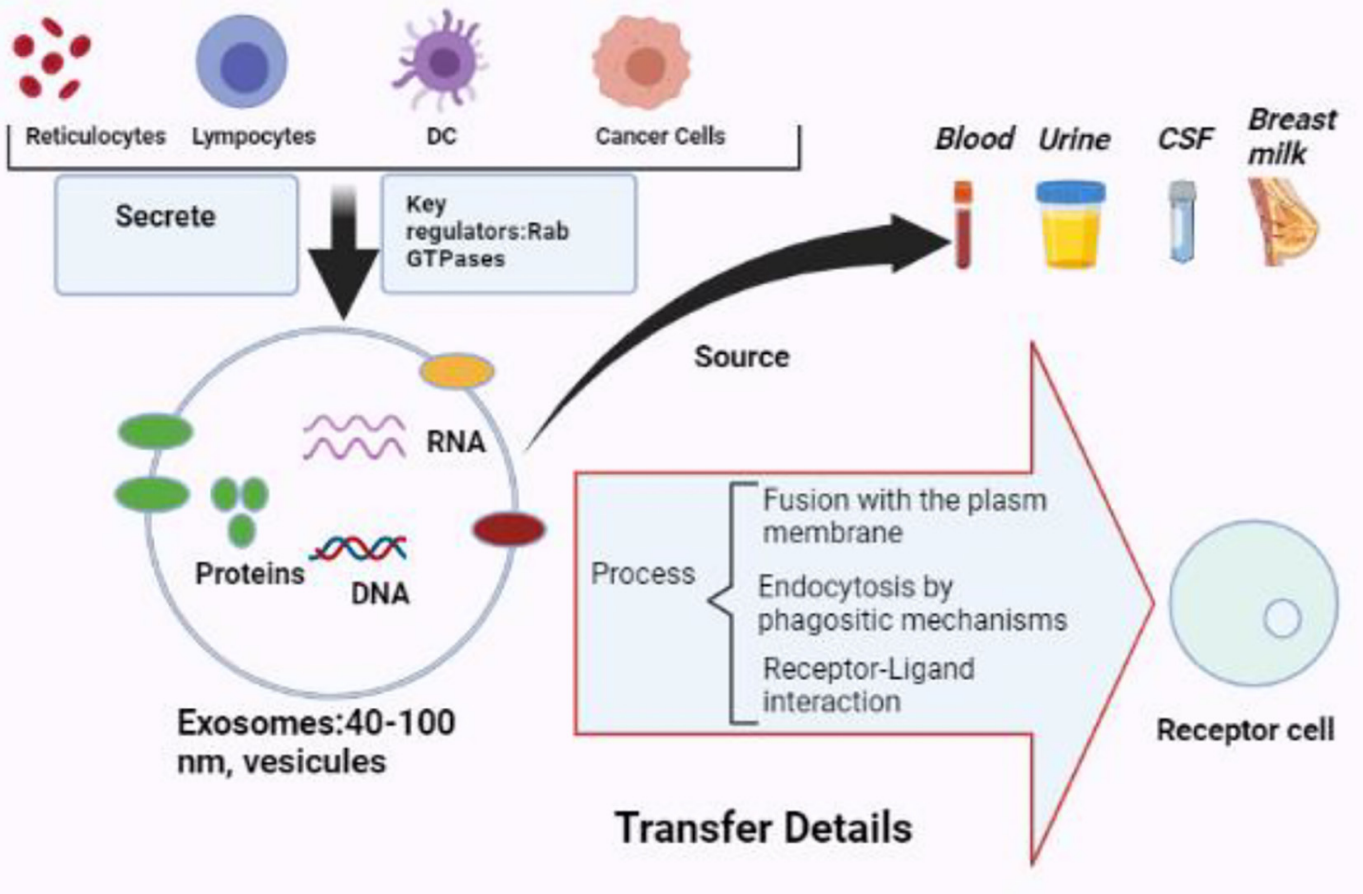
The biology of exosomes. Exosomes are vesicles with a diameter of 40 to 100 nm that may be produced by various cells and extracted from multiple bodily fluids. Exosomes may provide information to receptor cells *via* plasma membrane fusion, phagocytic endocytosis, or cell–receptor contact (BioRender.com was used to create this figure).

Exosomes are extracellular vesicles with a diameter of around 30–100 nm with a bilayer membrane (18, 19). Exosomes include several cargo types comprising proteins, lipids, enzymes, transcriptional factors, DNA fragments, mRNAs, micro-RNAs, and Long non-coding RNA (lncRNAs) (20, 21). Exosomes are released by different cell types, such as erythrocytes, platelets, lymphocytes, dendritic cells (DCs), adipocytes, fibroblasts, brain cells, stem cells, and cancer cells. Exosomes are detected in biofluids, including blood, plasma, urine, Cerebrospinal fluid (CSF), milk, amniotic fluid, malignant ascites, saliva, and synovial fluid (**Figure 1**). They play a significant part in the signals of normal and pathological processes in communication between cells and transporting substances such as proteins and RNAs from donor cells to recipient cells (16, 22, 23).

The biogenesis of exosomes occurs by inward budding of the plasma membrane that forms the endosome vesicle and the multivesicular bodies (MVBs). MVBs fuse with lysosomes, degrade or fuse with the plasma membrane, and create exosomes released from cells into extracellular space (22). Late endosomal structures containing dozens of Intraluminal Vesicles (ILVs) are known as MVBs, which are eventually transported to the trans-Golgi network for endosome recycling, delivered to lysosomes for degradation of all carried material, or fused with the plasma membrane and release exosomes into the extracellular space; this process is facilitated by Rab GTPase, such as RAB11 and RAB35, which release exosomes enriched with flotillin and other cell-specific proteins (24). Thus, exosome biogenesis and secretion require forming an endosomal-sorting complex required for transport (ESCRT) (25). ESCRT comprises four complexes (ESCRT-0, ESCRT-I, ESCRT-II, and ESCRT-III) and associated proteins (VPS4, Tsg101, and ALIX). ESCRT-0 sorts ubiquitinated cargo proteins into the lipid domain; ESCRT-I and ESCRT-II induce membrane deformation to form the stable membrane neck, and recruitment of the Vps4 complex to ESCRT-III drives vesicle neck scission and the dissociation and recycling of the ESCRT-III complex (26, 27).

A broad range of research has indicated an ESCRT-independent route in the exosome synthesis and carrier packing of lipids and related proteins, including tetraspanine (28). In opposition to ESCRT-sorted proteins, the loading of RNA into exosomes becomes lipid-mediated, reliant on self-organizing fat and carrier motifs. Specific nucleotide sequences show improved phospholipid bilayer affinity that relies on variables such as lipid morphology, hydrophobic changes, and physiologically concentrated sphingosine within rafting membranes (29, 30). Lipid rafts are plasma membrane subdomains loaded in cholesterol, sphingolipids, and attached proteins of glycosyl-phosphatidylinositol (GPI) whose connection with proteins or compounds may help their release by the use of exosomes (31, 32). The availability on the limiting membrane of ceramide, lysophospholipid, and glycosphingolipid molecules promotes the impulsive entry method to create ILVs (33). The ceramide changes in the existence of ceramidase and sphingosine kinase into sphingosine and sphingosine phosphate and the continual stimulation of 1-phosphate sphingosine receptors on the limited membrane facilitate the kind of tetraspanin into ILV. Thirty-three, three-four tetraspanin is a cell surface protein superfamily member with four transmembrane domains. Tetraspanin organizes membrane microdomains termed TEMs with a broad range of transmembrane and cytosolic signaling proteins (34). As the first tetraspanin, CD63 works in ESCRT-independent ILV creation. Interestingly, the lack of an ESCRT machine did not prevent the formation of MVB vesicles in mammalian cells but led to decreased cargo processing and changes in ILV numbers and sizes (35), indicating that exosome biogenesis could be a synchronized process that involves ESCRT-dependent and ESCR-independent pathways. The methods of exosome penetration into recipient cells have not been adequately explored. Nevertheless, it has been demonstrated that, according on the recipient cell type, exosomes penetrate target cells *via* fusion with the plasma membrane, macropinocytosis, phagocytosis, and clathrin-dependent endocytosis.

## 3 The Roles of Exosomes in Glioma Development

**Figure 2:**
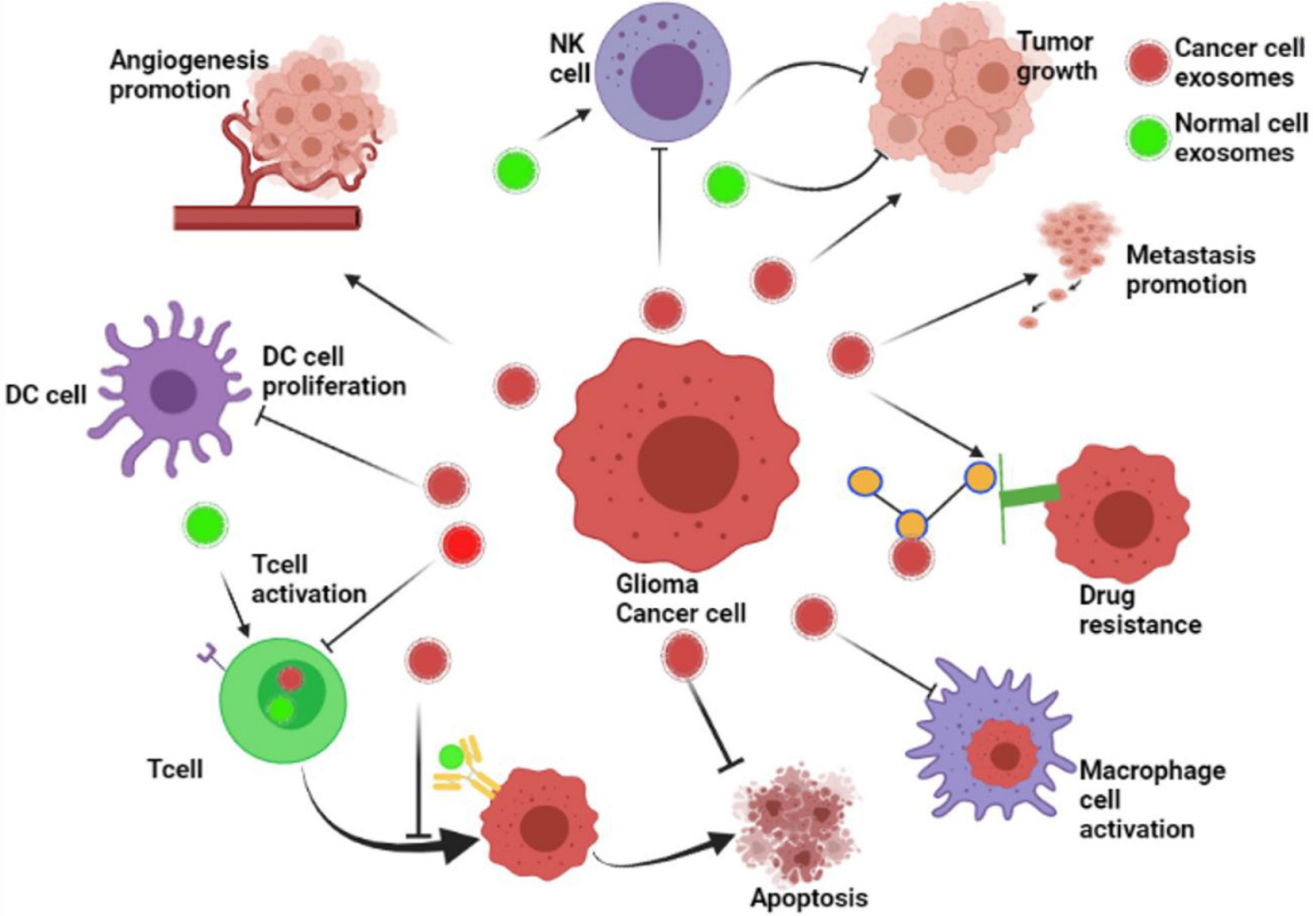
The roles of exosomes in glioma’s development. The role of exosomes in cancer progression. Cancer cell–derived exosomes influence the formation of the pre-metastatic microenvironment, tumor growth and advancement, immune escape, angiogenesis promotion, stopping of apoptosis, drug resistance, and metastasis. Besides, exosomes from healthy cells, including dendritic cells (DCs), B cells, and T cells, play a role in the inhibition of tumor growth (BioRender.com was used to create this figure).

Exosomes play a critical function in cell–cell communication by transporting bioactive materials from the donor cells to the receiving cells (36). Many pieces of research have revealed that cancer cells release more exosomes, both locally and at a distance, to share information with other cells (37). Cancer-derived exosomes contribute to pre-metastatic milieu creation, tumor development, progression, immune evasion, angiogenesis, anti-apoptotic signaling, and treatment resistance throughout their bioactive cargo. Meanwhile, healthy cell exosomes such as DCs, B cells, and T cells significantly prevent tumor growth (38). To date, many miRNAs, lncRNAs, and proteins have played a vital role in the development of cancer. Therefore, exosomes may play a dual function in controlling, preventing, or encouraging the development of cancer, depending on their cell of origin and bioactive cargo (**Figure 2**).

It has been reported that over 48 h, a single glioma cell secretes around 10,000 EVs (39). Glioma cell exosomes transport different chemicals than regular glial cell exosomes (40). Cancer effectors such as a mutant oncoprotein, oncogenic transcripts, and miRNAs are among the elements involved in tumor formation (28). In addition, exosomes enhance communication between cancer cells and the stromal cells surrounding them, resulting in either the tumorigenesis of apparently normal cells or a change in their behavior, which offers a favorable environment for the tumor to grow (41).

Angiogenesis is a critical event in glioma development, and glioma-delivered exosomes have been reported to play an essential role in this important event of glioma progression. For instance, a study that aimed to determine the processes of the glioma cell–affected angiogenesis noted that glioma cells might stimulate angiogenesis by transferring Linc-CCAT2 to endothelial cells through exosomes (42). Also, Lang HL et al. showed that gliomas stimulated angiogenesis by producing exosomes with high linc-POU3F3 (43); linc-POU3F3 has been reported to be involved in the development of glioma (43). On the other hand, a study showed that exosomes generated from hypoxic GBM cells relative to normoxic circumstances are effective inducers of angiogenesis *in vivo*. Moreover, they do the same *in vitro via* endothelial cell regulation, which increases tumor progression (44). Also, a study conducted by Xu Sun et al. noted that exosomes generated from glioma stem cells (GSCs) enhance endothelial cells’ angiogenic capacity through the miR-21/VEGF signaling pathway (45).

Moreover, different studies have revealed that exosomes are also involved in promoting and facilitating metastasis, which is a significant event in the progression and development of various cancers, including glioma. For example, Q. Cai et al. showed that miR-148a carried by exosomes facilitated cancer cell proliferation and metastasis by directly targeting CADM1 to trigger the STAT3 pathway (46), which plays an essential role in the metastasis of different cancer types, including glioma (47). Also, Zhanjun Ma et al. found that U251 cell–derived exosomes promoted metastasis-related proteins such as MMP-2 and MMP-9 (48), which facilitated the development of glioma. Moreover, another study also showed that the exosome EpCAM promotes the metastasis of glioma by targeting the CD44 signaling molecule on the surface of glioma cells; this exosome influenced the progression of glioma (49).

On the other hand, another study by Karma R Pace et al. discovered that exosomal L1CAM enhances motility, proliferation, and invasion in GBM cells, adding to the intricacy of how exosomal L1CAM promotes cancer cells not just through soluble ectodomains but also by exosomes (50). Besides, by directly targeting FBXW7 and DKK3, Gang Peng and his colleagues found that exosomal miR−25−3p significantly promoted the proliferation and migration of glioma cells (51). Furthermore, a recent study revealed that exosomal microRNA-671-3p increases cell proliferation in glioma by directly targeting CKAP4; this affected the proliferation and significantly increased glioma cell migration, facilitating glioma growth (52). Epithelial–mesenchymal transition is another vital factor that plays a significant role in glioma progression, and exosomes have been reported to be involved in glioma’s EMT. For instance, a study showed that exosomal microRNA-708 repression increases cell proliferation and EMT in glioma *via* promoting the SPHK2/AKT/-catenin pathway (53). In addition, another study also discovered that TGF-1 treatment substantially increased miR-10b expression in GBM cells, and miR-10b upregulation increases GBM cell proliferation, migration, and EMT; in contrast, miR-10b deletion has an opposite impact (54). Conclusively, the above facts show that exosomes play a crucial role in many critical glioma occurrences, such as cell proliferation, metastasis, angiogenesis, and EMT. More details about the involvement of exosomes in the progression of glioma are presented in **Table 1**.

**Table 1:** Exosomes that are involved in glioma.

Exosomes	Target	Outcome	References
miR-9	MYC and OCT4	Promotes tumorigenesis and angiogenesis	(55)
miR-1238	EGFR-PI3K-Akt-mTOR	Promotes proliferation, migration, and TMZ resistance	(56)
miR-148a	CADM1/STAT3	Promotes proliferation and metastasis	(46)
miR-1587	Inhibiting NCOR1	Increases tumorigenicity	(57)
MicroRNA-148a-3p	Inhibiting ERRFI1	Promotes tumor angiogenesis	(58)
MicroRNA-155-3p	Targeting Six1	Promotes glioma progression and temozolomide	(59)
MicroRNA-6807-3p	Targeting downstream DACH1	Promotes the tumorigenesis of glioma	(60)

TMZ, trimetazidine.

## 4 The Application of Exosomes as Future Biomarkers and Therapeutic Options for Glioma

### 4.1 Exosomes as Biomarkers for Glioma

GBMs are histologically varied tumors composed of many and different cell types. Notably, managing GBM is a significant problem for neurosurgeons. The current standard of care for GBM is magnetic resonance imaging (MRI) followed by surgery or brain biopsies. Both of these methods, however, have drawbacks (61).

Additionally, it is difficult to differentiate between tumor recurrence and postsurgical necrotic areas without histological investigation. On the other hand, collecting histology samples through immediate surgery or biopsies is time-consuming and hazardous, owing to the accompanying surgical risks. In addition, this is a one-time procedure with uncertain reliability due to the tumor’s heterogeneity, so using exosomes would be the best option to overcome those drawbacks in the future (62). Different significant studies have been conducted to assess if exosomes can be used as diagnostic and prognostic biomarkers; for example, a study reported that exosomal miR-181 could be a potential biomarker for human glioma at its initial stage. Interestingly, detecting miR-181 high rates may be a promising alternative for diagnosing glioma, evaluating the World Health Organization grade of tumors, and guiding in glioma medical management (63). Subsequently, another research also noted that exosomal miR-124 was involved in different glioma events such as promoting angiogenesis and chemoresistance, and the same study also found that exosomal miR-124 is a potential diagnostic marker of glioma (64). However, the researchers did not stop to continue to do more in-depth studiess on how exosomes are suitable candidates for the diagnosis and prognosis of glioma. That is why a study conducted by Fengming Lan and his colleagues, which had the primary purpose of determining both the diagnostic and prognostic values of exosomal miR-301a in patients with glioma, reported that exosomal miR-301a could show both cancer status and some changes with pathological changes in human glioma, which made miR-301a an excellent candidate for the diagnostic and prognostic biomarkers of glioma (65).

Furthermore, another study reported that people who suffer from glioma had increased levels of miR-205 and had improved overall survival rates compared to those who had decreased expression levels of miR-205. In addition, the authors of the same study concluded that the exosomes mentioned above could significantly be a prognostic biomarker for patients with advanced pathological glioma grades; it can also be used as a potential biomarker for the same cancer (66). Moreover, another research that aimed to determine the predictive and diagnostic significance of exosomal miR-221/miR-222 found that the increased positive plasma of both miRNAs was paired with low survival rates. They also found that both miR-221 and miR-222 are valuable tools to reveal glioma (67). Subsequently, a study that aimed to determine the clinical significance and diagnostic value of miR-128 reported that the diagnostic odds ratio was considerably high; this meta-analysis study highlighted that miR-128 could be a potentially non-invasive biomarker of glioma (68). In the meantime, the analysis of miR-21, miR-222, and miR-124-3p in serum exosomes in persons with glioma could provide a minimally invasive and revolutionary method to the differential diagnosis of glioma at their initiation in the brain and forecast glioma grade and non-glial metastases before surgery (69). Furthermore, it has also been revealed that the high rate of small RNU6-1, together with miR-320 and miR-574-3p, was found to be correlated with GBM IV diagnosis (70); more details are shown in **Table 2**.

**Table 2:** Various available exosomes considered as biomarkers for glioma.

Exosomes	Roles in Glioma	References
CircNFIX	CircNFIX augments TMZ resistance in glioma by sponging miR-132, indicating a possible prognostic biomarker.	(71)
HOTAIR	The levels of HOTAIR in tumor samples are much higher than in normal samples, and this research indicated that HOTAIR might be utilized as a prognostic and diagnostic biomarker for glioma.	(72)
miR-181	Detection of the level of miR181 family members may be a potential method for glioma diagnosis, determining the tumor WHO grade, and guiding clinical treatment.	(63)
miR-301a	Patients with an advanced pathological grade (III or IV) and an increased serum exosomal miR-301a level revealed a more prolonged overall survival than those with a lower level; this made mi-301a an ideal prognostic and diagnostic biomarker for glioma.	(65)
miR-205	Serum miR-205 levels were significantly increased in postoperative samples over preoperative samples and were reduced again during glioblastoma recurrences. miR-205 expression is a novel and valuable biomarker for diagnosing glioma and a prognostic factor for those with a tumor at an advanced pathological grade.	(66)
miR-128	circulating miR-128 is a promising non-invasive biomarker for diagnosing glioma.	(68)
miR-21, miR-222, and miR-124-3p	miR-21, miR-222, and miR-124-3p in serum exosomes of patients affected by gliomas can provide a minimally invasive and innovative tool to help the differential diagnosis of gliomas at their onset in the brain and predict glioma grading and non-glial metastases before surgery	(69)

TMZ, trimetazidine.

By employing quantitative real-time PCR, Tan et al. determined HOTAIR expression in serum from 43 GBM patients and 40 controls. It was found that HOTAIR levels were substantially high in serum samples from GBM patients than with matched controls. In addition, the expression of HOTAIR was found to be strongly associated with high-grade brain cancers, and Pearson’s correlation analysis revealed a moderate association between serum and tumor HOTAIR levels. As a result, serum HOTAIR can predict and diagnose GBM (72). Besides, another study performed on glioma patients found that exosomal CircNFIX levels were high in the serum of the patients resistant to TMZ, and CircNFIX high levels predicted poor prognosis, making it a potential prognostic biomarker for gliomas (71). Moreover, Chandran and colleagues conducted a study to explore extracellular plasma vesicles, and their research showed that Syndecan-1 is a critical biomarker for differentiating low- and high-grade gliomas (73). The tumorigenic epidermal growth factor receptor III is frequently overexpressed in high-grade glial brain tumors (EGFRvIII) (74). Combining both EGFRvIII and exosomes might be a good platform to create new effective biomarkers for glioma. For example, a study that had the main purpose of establishing a clinically adaptive protocol as a non-invasive diagnostic tool for *EGFRvIII* detection through serum exosomes found that the accuracy of EGFRvIII detection through exosomes was 80% for tissue EGFRvIII expression with an overall sensitivity and specificity of 81.58% and 79.31%, respectively (75). This study concluded that using exosome-based liquid biopsy to assess EGFRvIII expression for high-grade glioma diagnosis is extremely promising. It may assist in distinguishing high-grade gliomas from infective demyelinating illnesses with comparable radiological features.

Different advances have been made to produce new precision therapeutic and diagnostic tools for different types of cancer, including glioma. Theranostics is one of the new fast-expanding disciplines that combines nanotechnology’s unique possibilities with customized medicine for substantially improving effectiveness with decreased off-target impacts by providing therapy for targeted tissues (76). In 2021, Batla S. Al-Sowayan et al. mentioned that diagnostic and forecast biomolecular profiles may be developed utilizing nanogenomics and artificial intelligence for breast tumors based on the exosome packed content instead of free circulating miRNA and other biomolecules, which is an integrated approach toward the discovery of practical therapeutic and diagnostic tools for various types of cancer including glioma (77). Moreover, another study found that using both learning machines and nanofluids that encapsulate exosomes distinguished cancer and precancer mice from healthy controls and pancreatic cancer patients from healthy controls. Furthermore, deep sequencing is based on new and sophisticated technologies that enable billions of nucleotides to be sequenced in one run. Using this technology is crucial in discovering not only therapeutic agents but also biomarkers for glioma. For example, using deep sequencing technology, Saeideh Ebrahimkhani et al. noted that serum exosomal miRNA signatures could accurately diagnose GBM preoperatively; this makes this exosomal miRNA signature a potential diagnostic biomarker for glioma (78). Altogether, the above-mentioned facts show that exosomes are potential candidates to be glioma biomarkers.

### 4.2 Exosomes as Therapeutic Agents for Glioma

Cancer treatment is one field that is developing at a very significant speed (17). Additionally, researchers realized that exosomes might also be involved in the treatment of glioma. For example, a study, which had the primary aim to evaluate whether marrow stromal cell (MSC) exosomes can be used as a vehicle for the delivery of anti-tumor miRNAs, found that transfected MSCs with miR-146b plasmid expression harvested MSC-released exosomes and intra-tumor injection of exosomes extracted from miR-146-expression and MSCs significantly decreased glioma development in the primary brain tumor in the used mouse model (79). Chemoresistance is another issue in treating different cancers, including glioma. Exosomes have been linked positively to this issue by restoring chemosensitivity. For example, the transmission of anti-miR-9 to resistant GBM cells restored the multidrug transmitter’s function and attuned the GBM cells to TMZ, as shown by increased cell death and caspase activity. The findings showed the role of MSCs in the empirical distribution of synthetic anti-miR-9 in overcoming GBM cells’ chemoresistance (80). Besides, exosomal transfer of long non-coding RNA SBF2-AS1 enhances chemoresistance to molozonide in GBM by secreting the oncogenic LincSBF2-AS1-enriched exosomes (81). Yin J et al. showed that exosomal miR-1238 contributes more to the modulation of gained GBM chemoresistance; exosomal miR-1238 can induce the chemoresistance microenvironment of the tumor (82).

Furthermore, exosomes have been reported to be outstanding candidates to stop the tumor from spreading in various cancers, including glioma. For example, a study that aimed at determining the clinical roles and the regulatory mechanism of miR-454-3p in glioma found that regaining the expression of miR-454-3p inhibited significantly different features such as cell proliferation, migration, invasion, and autophagy in glioma, which made miR-454-3p be considered as a glioma tumor suppressor and a treatment agent for glioma (83). Moreover, another study reported that exosomal miR-451 impeded the proliferation, invasion, and apoptosis of GBM cells, and this study concluded that exosomal miRNA-451 could act as a tumor suppressor in human gliomas (84).

In the continuing movement of discovering new treatments for glioma, various studies have been conducted and showed that some exosomes could be potential therapeutic targets in glioma, which is regarded as an excellent cancer treatment approach. For instance, a study reported that the repression of miR-10b in the human glioma mouse model results in a more significant tumor progression decrease. Furthermore, their study briefly affirms the essential role of miR-10b in glioma initiation, unveiling the novel mechanism of miR-10b-mediated control and showing the likelihood of its potential use as a therapeutic target in glioma (85).

**Figure 3:**
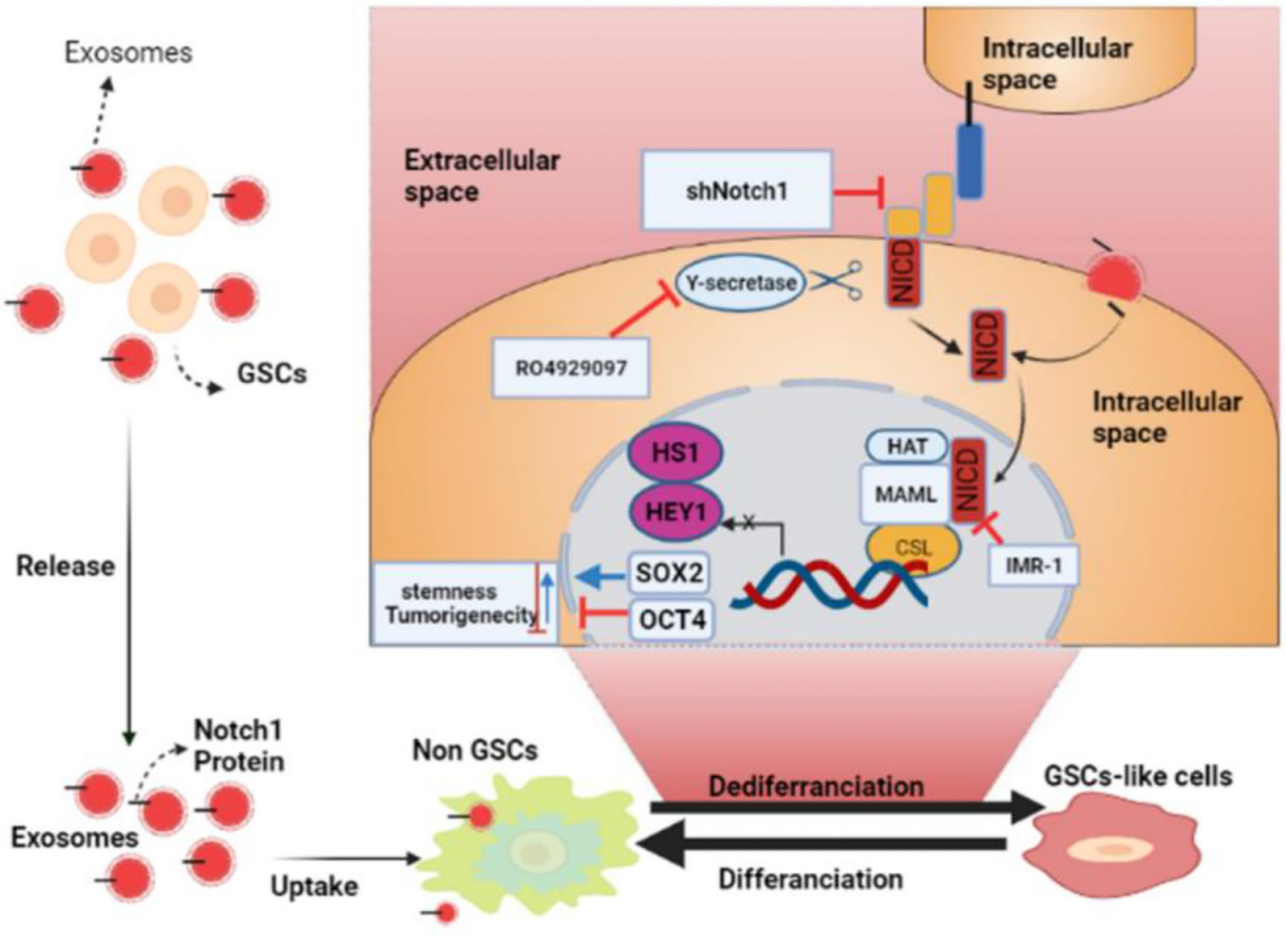
The roles of glioma stem cell (GSC) exosomes in glioma. GSC exosomes improve the stemness and tumorigenicity of non-GSC glioma cells by conveying Notch1 protein *via* the Notch1 signaling pathway (BioRender.com was used to create this figure).

It has also been found that exosomal miR-34 can be used as a tumor suppressor for glioma by targeting two essential genes, c-Met and Notch (86). Also, it has been found that miR-146a inhibits glioma growth by directly targeting and stopping the Notch1 pathway activity (87). Lei Yu et al. also reported that mesenchymal stem cells that transmitted miR-199a to the glioma cells by exosomes suppressed different important glioma features, such as cell proliferation, invasion, and migration. Besides, high expression of miR-199a in mesenchymal stem cells that restored TMZ chemosensitivity miR-199a also suppressed glioma by downregulating AGAP2 (88). Another study revealed that miR-1246, detected in the cerebrospinal fluid in patients suffering from GBM, could be a diagnostic biomarker. This study also noted that targeting microRNA-1246 may lead to anti-tumor immunotherapy (89). Also, another study reported that GSCs produced exosomes carrying Notch1 protein; when these exosomes were absorbed into non-GSC glioma cells, Notch1 transferred from GSC exosomes activated the Notch1 signaling pathway, increasing the stemness and tumorigenicity of these non-GSC glioma cells. In addition, GSC exosomes serve as information carriers, facilitating the dedifferentiation of non-GSC glioma cells into GSCs by conveying Notch1 protein and activating Notch1 signaling, maintaining the dynamic equilibrium state of GSCs in the tumor microenvironment. GSC exosomes and the Notch1 signaling pathway targeted to harm GSCs might be a unique method for GBM eradication that needs further exploration (**Figure 3**). The above facts show that continuous studies will improve the likelihood of exosomes to be used as future glioma treatments (90). More details concerning the roles of exosomes in the treatment of gliomas are summarized in **Table 3**.

**Table 3:** Exosomes that are involved in glioma treatment.

Exosomal content	Experimental Design	Mechanism	Outcome	References
miR-21 sponge construct	*In vitro* and stereotaxically injected into a rat model	Downregulates miR-21 and upregulates miR-21 target genes (PDCD4 and RECK)	Reduces tumor volume	(91)
miR-34a	*In vitro* and subcutaneously injected into a rat model	Downregulates MYCN	Suppresses GBM cell growth, invasion, migration, and tumorigenesis and enhances chemosensitivity of the GBM cells to TMZ	(92)
miR-375	*In vitro* and a rat model	Suppresses SLC31A1	Promotes apoptosis and suppresses proliferation, migration, and invasion	(93)
microRNA-7-5p	A subcutaneous tumor model and tumor metastasis model of nude mice were established to validate the *in vitro* findings	Inhibits the activity of the EGFR/PI3K/Akt signaling pathway	Suppresses the proliferation, migration, invasion, and microtubule formation	(94)
miR-29a-3p	*In vitro* and *in vivo*	Target ROBO1	Stops migration and VM (vasculogenic mimicry) formation in glioma cells	(95)
miR-15a and miR-92a	*In vitro*	Inhibit the activity of the PI3K/AKT/mTOR signaling pathway	Inhibit cell migration and invasion of glioma cells	(96)
miR-199a	*In vitro*	Downregulates AGAP2	Suppresses tumor proliferation, invasion, and migration	(88)
miR-454-3p	*In vitro*	Targets ATG12	Tumor suppressor in glioma	(83)
microRNA-512-5p	*In vivo* and *in vitro*	Targets JAG1	Inhibition of glioblastoma progression	(97)
microRNA-133b	*In vivo* and *in vitro*	Inhibits EZH2 and the Wnt/β-catenin signaling pathway	Represses glioma cell proliferation, invasion, and migration	(98)

PDCD4, Programmed Cell Death 4; RECK, Reversion-inducing Cysteine-rich Protein with Kazal motifs; MYCN, MYCN proto-oncogene, bHLH transcription factor; SLC31A1, Solute Carrier Family 31 Member 1; ROBO1, Roundabout Guidance Receptor 1; AGAP2, ArfGAP With GTPase Domain, Ankyrin Repeat And PH Domain 2; ATG12, Autophagy Related 12; JAG1, Jagged Canonical Notch Ligand 1; EZH2, Enhancer Of Zeste 2 Polycomb Repressive Complex 2 Subunit; EGFR, Epidermal Growth Factor Receptor; PI3K/Akt , Phosphoinositide-3 kinase/Akt; GBM, Glioblastoma; TMZ, Temozolomide.

#### 4.2.1 Exosomes as a Crucial Delivery Method of Various Therapeutic Agents to Glioma Tumors

**Figure 4:**
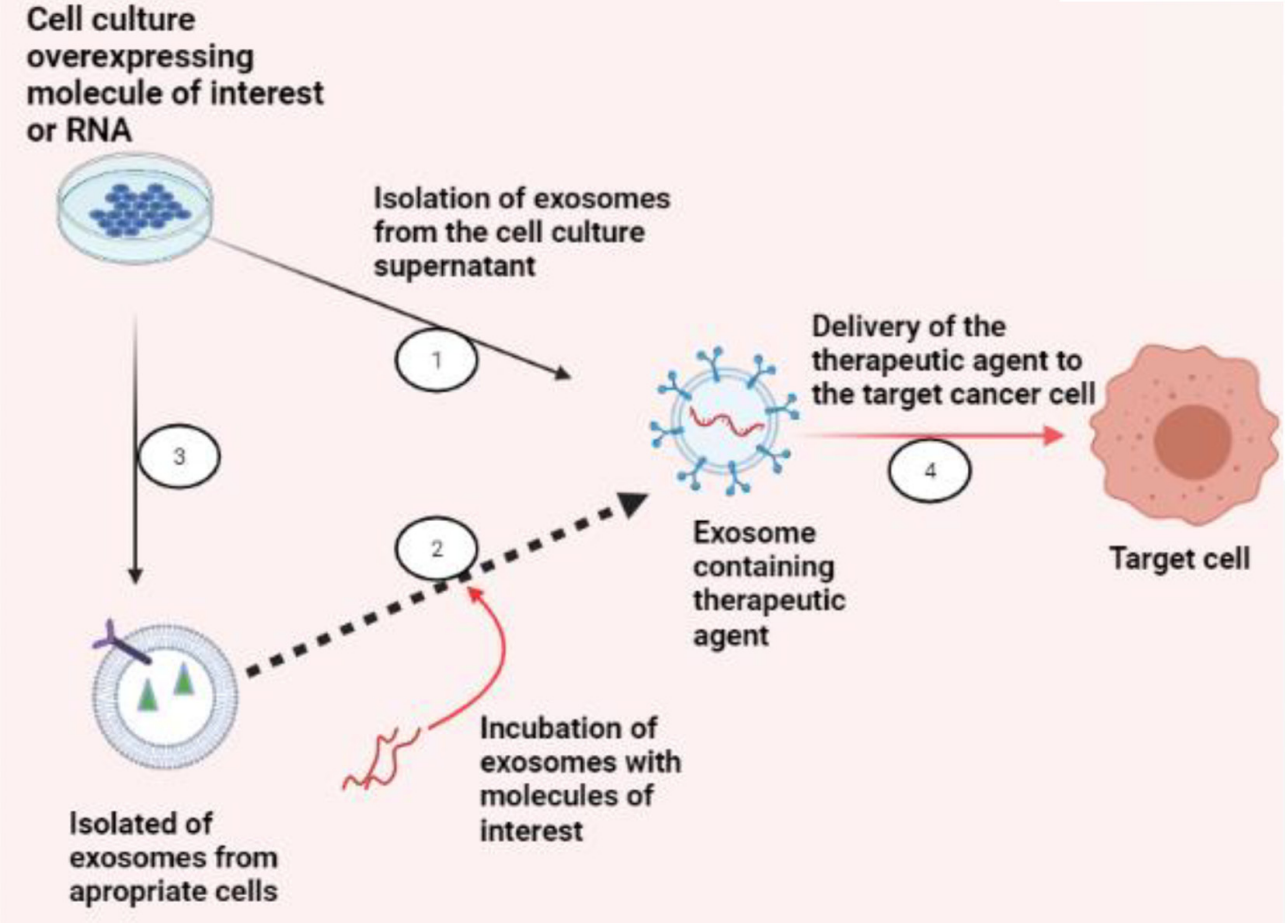
Exosomes as a therapeutic carrier. There are two strategies to load exosomes with therapeutic cargo, like RNA species for gene silencing in targeted cancer cells or small molecule compounds of concern: (1) endogenously, by collecting exosomes from cells overexpressing the molecule of interest, or (2) exogenously, by collecting exosomes from an appropriate cell culture that produces exosomes suitable for specific targeting and then incubating or electroporating the exosomes with the molecule of interest. (3) Once the exosomes are successfully loaded, (4) exosomes can be used for their respective therapeutic applications (BioRender.com was used to create this figure).

Drug loading may be done, whether endogenously or exogenously (**Figure 4**). By employing normal cell culture procedures, endogenous or passive loading is carried out by overexpressing the RNA species or molecule of interest. This passive loading is facilitated by the cell’s natural exosomal loading processes and results in exosomes that contain the medication before isolation. Exogenous or active loading starts with exosome collection. Then, it involves either co-incubation or electroporation of the exosomes with the drug, and afterwards, the exosomes can be safely delivered to the target cells (99).

To enhance the efficacy of cancer therapy, medicines must be delivered precisely to tumor cells. Clinically, medication delivery techniques based on nanotechnology are one of the most promising means to accomplish this task. Exosomes have been effectively utilized as medicinal and functional RNA delivery vectors for cancer treatments (100). Exosomes may be absorbed by cells and medicines such as therapeutic miRNAs and proteins that are securely transferred (101). For example, in 2018, Gang Jia and His colleagues firstly loaded super-paramagnetic iron oxide nanoparticles (SPIONs) and curcumin (Cur) into exosomes. They then conjugated the exosome membrane with neuropilin-1-targeted peptide by click chemistry to get glioma-targeting exosomes with imaging and therapeutic functions. When delivered to glioma cells and orthotopic glioma models, they observed that these modified exosomes could cross the BBB smoothly, and exosomes offered extraordinary outcomes for targeted imaging and treatment of GBM. SPION-mediated magnetic flow hyperthermia and Cur-mediated therapy also had a powerful anti-cancer effect when used together (102).

Interestingly, another study by Hamideh et al. showed that the administration of the exosomes carrying miR-21 into a glioma mouse model reduced the volume of the tumor (91). Moreover, Jessian L et al. also found that delivering the mesenchymal stem cell–derived exosomes to GBM loaded with anti-miR-9 increased the temozolomide chemosensitivity (80). Improving the loading capacity of an anti-cancer agent into exosomes is critical for enhancing anti-cancer medication delivery to glioma and keeping the proper drug dose in glioma tissues. Thus, several approaches, including electroporation, incubation, and chemical reagents, have been examined to increase the loading efficiency of therapeutic medicines into exosomes. Furthermore, the application of microfluidics to drug loading and delivery to cells has been researched, especially with the advancement of micro- and nanofabrication technologies (103). Recently, therapeutic loading into exosomes using microfluidics has received attention and progress from different researchers. For example, a recent study conducted by Thakur A et al. revealed that Exo-Load microfluidic device fully incorporated two BBB-impermeable anti-cancer drugs, DOX and PTX, into SF7761 stem cell–like GM-derived exosomes in the presence of saponin, a permeabilization agent, and shear stress in microfluidic channels. The sigmoid Exo-Load type outperformed the linear kind of Exo-Load in loading DOX into U251 GM-derived exosomes, indicating that Exo-Load-based drug loading into exosomes may be promising with future modification and optimization techniques. This study concluded that Exo-Load (microfluidic) device-based loading of anti-cancer drugs into exosomes and autologous uptake of EXO-DOXs might effectively suppress the proliferation of glioma cells (104). The facts mentioned above show that exosomes play an essential role in delivering different anti-cancer drugs, critical for managing glioma.

#### 4.2.2 Exosomes as Therapy Response Monitor in Glioma

Monitoring treatment is another essentially major step in different cancer treatments. As discussed earlier, exosomes play essential roles in various vital events involved in glioma; besides, exosomes monitor various therapies’ sensitivity in glioma. For example, Ailiang Zeng et al. found that exosomal miR-151a is not only a less invasive ‘liquid biopsy’ that may predict chemotherapy response, but miR-151a is also a promising therapeutic target for refractory GBM therapy (105). Moreover, *in vivo* studies verified MSC-derived exosomes’ ability loaded with miR-133b to inhibit glioma tumor growth, and MSC-derived exosomal miR-133b and the Wnt/β-catenin/EZH2 pathway could act as biomarkers for monitoring and prognosis in glioma therapy (106). Moreover, another research highlighted that exosomal miR185 and miR-205 are potential candidates to help clinically track different treatment responses in glioma (107). Recently, another study discovered that hypoxia-induced malignant GMs significantly increased MCT1 and CD147 expression, thereby facilitating calcium-dependent exosome secretion. Additionally, it was discovered that hypoxic GM-derived exosomes consisted of substantially increased levels of MCT1 and CD147, which could be quantified using non-invasive localized surface plasmon resonance and atomic force microscopy biosensors, demonstrating that they could serve as precise surrogate biomarkers for tracking metabolic reprogramming and malignant progression of glioma (108). Also, another recent study conducted by Chen Xu et al. used a TiO2-CTFE-AuNIs plasmonic biosensor to identify BIGH3 in exosomes produced from glioma cells to monitor the malignant evolution of glioma. Thus, TiO2-CTFE-AuNIs was reported to be capable of quantifying the dynamic change in exosomal BIGH3 in response to hypoxia and TMZ therapy. This allows the measurement of BIGH3 levels in parent GMs, revealing TMZ’s anti-cancer impact; this makes the biosensor mentioned above show significant promise for its applicability to the identification of predictive biomarkers in GM-derived exosomes for glioma liquid biopsy (109). Even though there is a great job done about using exosomes as diagnostic and prognostic biomarkers, there is a need for deep researches to prove that exosomes can be used to monitor various therapies in glioma, which can be used in daily clinical life. This will significantly increase patients’ treatment outcomes.

#### 4.2.3 Clinical Trials Related to Roles of Exosomes in Glioma Diagnosis and Treatment

Clinical trials help scientists and clinicians test diagnostic and treatment tools; this applies to other cancers, including gliomas. Different clinical trials have been performed to highlight the usefulness of exosomes in the treatment and diagnosis of various cancers such as pancreatic cancer (NCT02393703) and colorectal cancer. Even though there are not too many clinical trials dealing with the potential roles of exosomes in glioma, one recruiting clinical trial aims to determine if mir-10b expression levels are a fit candidate to be a prognostic and diagnostic marker (NCT01849952); the details on the mentioned clinical trials and others are presented in **Table 4**. The facts mentioned above show hope that exosomes are future diagnostic and treatment tools for glioma. However, there is still a need for more clinical trials to prove the use of exosomes’ roles as routine diagnostic and treatment agents for glioma.

**Table 4:** Examples of clinical trials done on the roles of exosomes as biomarkers or in treatment agents for glioma and other cancers.

Status of the Clinical Trials	Objectives	Condition of the Disease	Clinical Trial Identifier
Completed	Establishment of a signature of circulating microRNA as a tool to aid diagnosis of primary brain tumors in adults	Brain tumors	NCT03630861
Recruiting	Evaluating the expression levels of microRNA-10b in patients with gliomas	Astrocytoma, oligodendroglioma, oligoastrocytoma, and others	NCT01849952
Recruiting	Assessing blood and cerebrospinal fluid metabolomic profile in glioma patients	Glioma, glioblastoma multiforme	NCT03865355
Completed	Evaluating microRNAs as disease markers for central nervous system tumors in patients with neurofibromatosis type 1	Glioma, neurofibromatosis type 1	NCT01595139
Recruiting	Assessing the ability of exosomes in treating participants with metastatic pancreatic cancer with KrasG12D mutation	KRAS NP_004976.2: p.G12D, Metastatic pancreatic, adenocarcinoma, pancreatic ductal adenocarcinoma, stage IV pancreatic	NCT03608631
Recruiting	To characterize exosomal biomarker levels in patients with locally advanced rectal cancer undergoing neoadjuvant chemoradiation therapy	Rectal cancer	NCT03874559
Recruiting	Identification of new diagnostic protein markers for colorectal cancer	Colorectal cancer	NCT04394572

## 5 The Involvement of Exosomes in Glioma Immune Responses

Exosomes play a crucial function in tumor-immune cell cooperation. Besides, exosomes secreted by tumor cells carry tumor-specific antigens that, in extraordinary circumstances, enable and suppress the immune system and promote the proliferation, invasiveness, and chemoresistance of glioma. miRNAs released from the tumor-derived exosomes can regulate the differentiation and function of the immune cells. These tumor-derived exosomes have many physiopathological roles and act on a range of immune cells, including effector T cells, naturally occurring Treg cells, and natural killer cells synonymous with immune suppression and tumor progression (110, 111). Also, serum exosomes from GBM patients have been shown to cause M2 polarization in normal monocytes, indicating a tendency toward T-helper 2 cells (Th2). Th2 responses are considered unacceptable in tumor immunotherapy since they alleviate cytotoxic anti-tumor immune processes and help inhibit cell-mediated immunity (112). Microglia-derived exosomes often mediate essential immune responses to tumorigenesis, degeneration, and central nervous system infections (113). Antigen-dendritic cells induce T-cell activation upon incubation with genes with different expression levels (GDEs) and mediate cytotoxicity against *in vitro* (114). Hell Winkel and his colleagues observed that immunosuppressive phenotypes and elevated cytokine concentrations in exosomes extracted from tumors lead to decreased development of other cytokines, including interleukin 2 (IL-2) CD69, and T-cell function, obstructing the migration of lymphocytes and inhibiting the immunity of tumors (115). Exosomes derived from GBM GL26 cells reduced cytotoxic CD8+ T cells’ number and function, fostering tumor growth (116). GDEs help to classify peripheral blood monocytes into alternately triggered M2 tumor-supporting macrophages (117) and regulate the development of cytokines and mononuclear migratory ability mitogen-stimulated, healthy, peripheral blood cells.

## 6 The Involvement of Exosomes in Glioma TME

A glioma’s TME is incredibly diverse, comprising various cancer and non-cancer cells, such as endothelial cells, immune cells, glioma stem-like cells, and atrocities’ non-cellular elements, including the extracellular matrix (118, 119). The tumor microenvironment is progressively recognized as a strong promoter of glioma advancement, playing a leading role in controlling tumor growth (120). Therefore, exosomes have been viewed as a necessary two-way contact between the tumor and the tumor microenvironment (121). Different studies have been conducted to reveal the linkage between exosomes and the glioma’s microenvironment. For instance, a study reported that the miR-340-5p-macrophage virtuous cycle changed GBM development and TM (122). Over the last few years, studies suggested EVs produced by GBM cells interact with the activation of this tumor-supporting Tumor-Associated Macrophage (TAM) phenotype modulation. For example, a study showed that EVs derived from GBMs’ primary cultures had manipulated the TAM phenotype *in vitro*, converting it into an M2-like anti-inflammatory phenotype that resembles a tumor-supporting phenotype found in patients. Phenotypic changes included changed expression of a wide range of cell surfaces and enhanced release of cytokines such as interleukin-6 (IL-6) and vascular endothelial growth factor. In addition, GBM-derived exosomes mediated and enhanced macrophages’ phagocytic activity, enhancing the extracellular matrix’s deterioration and promoting the migration of tumor cells (117). It has been shown that exosomes, isolated from GBM cell line U87 and the GSCs, primarily target monocytes to cause the restructuring of the actin cytoskeleton and immunosuppressive phenotype M2, with the release of cytokines like MCP-3 and CXCL1 (123). Exosomes are among the many ways GBM cells interact with the tumor microenvironment to their advantage. Based on the researches mentioned above, it is very clear that exosomes play essential roles in glioma TME. However, different advanced studies are needed to deeply highlight the extended functions of exosomes in gliomas’ tumor microenvironment.

## 7 Limitations and Future Prospective

Exosome research in gliomas is a new and fast-developing area. It is clear that exosomes can be used to create therapeutic strategies to prevent and treat glioma growth and development. Nevertheless, many issues remain unresolved, such as the lack of consistency and consistency in exosome detection, isolation, and purification methods. **Table 5** summarizes the benefits and drawbacks of exosome isolation techniques. Second, the approach by which exosomes are taken up by recipient cells is unknown. Additionally, brain tissue collection is more complicated. Additional researches are necessary to thoroughly understand the pathophysiology of exosomes in gliomas and show exosomes’ involvement in the illness.

**Table 5:** Exosome’s isolation methods, advantages, and disadvantages.

Methods	Theory	Advantages	Disadvantages	References
Ultracentrifugation techniques	The required components are obtained according to the size and density differences for each element in the sample.	There is no need to mark the outer cut body to avoid cross-contaminations.	High cost, time-consuming, structural failure, aggregation, and lipoprotein separation is not conducted to downstream analysis	(124, 125)
Density gradient centrifugation	Usually used in combination with the overspeed centrifuge method	Improves the purity of exosomes	The high viscosity of sucrose solution will reduce the settling velocity of exosomes and lead to more time consumed.	(126, 127)
Size-based isolation techniques	Based on the size differences between exosomes and other components of a biological sample	Fast, simple, low-cost, and separated exosomes have complete structure and uniform size. Their biological characteristics will not be significantly affected.	Other particles of similar size are difficult to separate, resulting in reduced purity.	(128)
Ultrafiltration	Ultrafiltration membranes with different molecular weight cutoffs were used to separate the samples selectively.	The sample cost is low, the concentration efficiency is high, and the activity of the exosomes is not affected.	Low purity and poor binding of the exosomes to the ultrafiltration membrane resulted in a low recovery rate.	(129)
Immunoaffinity chromatography	The specificity of antibodies and the ligand is combined to separate the required exosomes from heterogeneous mixtures.	The sample size needed is small. It can be used to qualitatively and quantitatively detect exosomes. This method has strong specificity, high sensitivity, high purity, and high yield.	The preservation condition of the exosomes obtained by this method is harsh. It is not suitable for large-scale separation of the exosomes. The non-specific interference adsorption of matrix produces interfering proteins, which limits the broad application of this method.	(130)

## 8 Conclusions

Exosomes are a novel mode of cell communication that facilitates communication between parent and target cells and changes the tumor microenvironment, promoting cancer progression. Recent research has highlighted their involvement in the pathways through which gliomas develop, infiltrate surrounding tissue, create resistance to therapy, and spread throughout the body. Continuously studying exosomes will alter and complement current understandings of carcinogenesis and development, thus contributing to a complete understanding of tumor-related molecular processes. It will also help discover efficient biomarkers and targeted tumor therapies using exosomes, improving efficacy and medication usage for glioma. However, more deep studies are needed that incorporate new advanced technologies such as machine learning, scRNA-seq, and high-throughput screening to enhance the characterization of exosomal drugs as carriers to get more reliable therapeutic and diagnostic results. In addition, more clinical trials are needed to prove exosomes’ usefulness as future daily therapeutic agents and biomarker tools for gliomas and other types of cancers.

## Author Contributions

XiaobW designed and drafted the manuscript. XingbW, JW, YH, FL, XinW, LY, and ZL discussed and revised the manuscript. All authors read and approved the final manuscript.

## Funding

This study was supported by the Clinical Medicine Science and Technology Innovation Project of JINAN (No. 202019020).

## Conflict of Interest

The authors declare that the research was conducted in the absence of any commercial or financial relationships that could be construed as a potential conflict of interest.

## Publisher’s Note

All claims expressed in this article are solely those of the authors and do not necessarily represent those of their affiliated organizations, or those of the publisher, the editors and the reviewers. Any product that may be evaluated in this article, or claim that may be made by its manufacturer, is not guaranteed or endorsed by the publisher.

## Abbreviations

GBM, glioblastoma; TMZ, trimetazidine; EMT, epithelial–mesenchymal transition; MDSCs, myeloid-derived suppressor cells; MCP-3, monocyte chemotactic protein 3; CXCL1, chemokine (C-X-C motif) ligand 1; GSCs, glioblastoma stem cells; TME, tumor microenvironment; EMT, epithelial–mesenchymal transition; DNA, deoxyribonucleic acid; RNA, ribonucleic acid; HBMEC, human brain microvascular endothelial cell; CADM1, cell adhesion molecule 1; STAT3, signal transducer and activator of transcription 3; CAB39, calcium-binding protein 39; AMPK, prime-AMP-activated protein kinase; FBXW7, F-box and WD repeat domain containing 7; DKK3, Dickkopf homolog 3; BBB, blood–brain barrier; circNFIX, circular RNA nuclear factor I X; SPION, superparamagnetic iron oxide nanoparticles; EZH2, enhancer of zeste homolog 2; GDEs, genes with different expression levels; EVs, extracellular vesicles; MCP-3, monocyte chemotactic protein; AGAP-2, Arf-GAP with GTPase; ANK repeat, and PH domain–containing protein 2, TiO2-CTFE-AuNIs, titanium dioxide (TiO_2_) columnar thin film (CTF), coupled with gold nanoislands (AuNIs); BIGH3, beta Ig-H3.
